# Migration and Workforce Planning in Medicine with Special Focus on Anesthesiology

**DOI:** 10.3389/fmed.2017.00111

**Published:** 2017-07-26

**Authors:** Jannicke Mellin-Olsen

**Affiliations:** ^1^Baerum Hospital, Sandvika, Norway

**Keywords:** brain drain, migration, global health, workforce, recruitment

## Abstract

Counting health personnel and defining migration is more complicated than one should think at first glance. Migrating health workers are not a homogenous group, and many factors cause people to migrate—not only low wages but also lack of professional development possibilities, poor job satisfaction, outdated equipment, unsafe environment, and more. The opposite factors encourage people to stay. Many countries, including high-income countries benefit from remittances from migrating individuals. The World Health Organization has installed a code of Practice on the international recruitment of health workers. Although member countries have committed to follow this Code, it is not widely adhered to. Planning for the future is difficult, also because there are so many unknown factors related to the development of health-care levels, policies, inflow and outflow and more. Action must be taken in both donor and receiving countries. In anesthesiology, there is a huge workforce deficit globally. The world would need 136,000 additional physician anesthesia providers today to achieve an absolute minimum of five per 100,000 population. This will not happen unless all countries follow those that already have taken proactive steps in leading the direction forward. Anaesthesiology Society involvement is crucial.

## Background

In 2005, Egger Halbeis and colleagues attempted to describe the anesthesia workforce in Europe (1). It became clear that such an attempt is more complicated than at first sight. For instance, the simple question “how many anesthesiologists are there in one country” cannot easily be answered: Are we talking about the number of registered anesthesiologists? This causes problems, as some are registered in several countries, but do not work in all of them. Do we include retired colleagues or only those active? Do we include only specialists, although in some countries, trainees are doing a major bulk of the workload? How do we count colleagues that work full-time versus part-time? etc.

## Defining “Foreign”

OECD has described the problem in depth (2). Migration patterns can be based on nationality. But then, the foreigners disappear from the statistics as soon as they are naturalized. In several OECD countries, many people who were born and raised in a country hold a different nationality. Hence, there is no systematic link between migration and nationality. An alternative would be to look at “foreign” as a function of the place of birth. If the country of birth differs from the country of residence or citizenship, it means that the person crossed a border at some point. But some of these persons arrived as small children, often with their families. Others came to attend medical school and have stayed on after graduation, and most people would regard this group as different from those who moved as small children. A third alternative would be to identify “foreign” as a function of the country where the education took place. But this creates other difficulties: doctors who were fully licensed in one country but had to redo all or part of their postgraduate training to get fully registered in their new host country could be identified as trained in that host country. In addition, some countries that do not have their own medical schools have agreements with other countries to train their doctors. In addition, there are quite a number of international medical students going abroad to get their medical education but return to their home country after graduation. Are they foreign? Are they migrants?

For example, in Austria, there were 14% foreign born medical doctors in 2011, but the number of foreign trained was 4% and foreign national citizens 8%. This illustrates that unless all subpopulations are identified, the data will hardly cover the populations in question entirely, and it makes simple statements hard to make.

Other illustrative examples are UK and Luxembourg. Both countries depend on foreign medical doctors at a similar level—35–40% of the total medical workforce. The impact in source countries is very different—in 2013, there were 91,000 internationally trained medical doctors in the UK and 605 in Luxembourg (3). Other countries with high dependency on foreign trained doctors are countries like New Zealand, Ireland, and the USA, whereas countries like Poland, Austria, and France to a large extent take care of their own training (4).

## Pattern of Physician Migration

The PROMeTHEUS/World Health Organization has defined health professional mobility as “any movement across a border by a health professionals after graduation with the intention to work, that is, deliver health-related services in the destination country, including during training periods” (3). A problem with this definition is that it includes false positive for nationals went abroad for training and returned. The World Bank has highlighted the problems with definition in their report, saying that “According to the “Recommendations on Statistics of International Migration” by the United Nations Statistics Division (1998), “long-term migrants” are persons who move to a country other than that of their usual residence for a period of at least 1 year, so that the country of destination effectively becomes their new country of usual residence” (5). However, they point out that not all countries operate with the duration of 1 year.

Nevertheless, the latest figures by the World Bank show that the top migration countries of physicians were in 2002 by numbers India, UK, and the Philippines (5). Emigrants calculated as percentage of the total numbers of physicians trained in the countries, Grenada and Dominica were the worst, both more than 97%. Of the high-income countries, as defined by the WHO, the worst were Iceland at 25.2% and Ireland at 24.6%.

Migration is often related to the concept “brain drain.” It leads to lack of workforce and eventually, to system collapse. Countries spend finances on education only to lose return on investment, as some sort of reverse/perverse subsidy to richer countries. When the WHO last focused on the health workforce in 2006, Africa had 25% of the world’s burden of disease, but only 1.7 share of the health personnel in the world (6). Already by 2015, poor countries annually spent USD 500 million to train doctors and nurses who move to the EU and USA (7). There are more African researchers living in the USA than in all of Africa. Ironically, after importing the brains of Africa, high-income countries in return give aid to counteract brain drain, it has been estimated that there is USD26 billion worth of foreign expertise in Africa (5).

However, it would be too simplistic to indicate that this is a total drain. Many of the donor countries receive remittances from the emigrants, and this sometimes is important for the country’s economy. In 2015, India received 72 billion USD, and China received 64 billion USD as remittance from the emigrants. France received 24.6 billion USD. For Tajikistan, the remittances accounted for 41.7% of the GPD in 2014 (8). The Philippines train health personnel for export as part of the strategy to raise money for the country (9).

Migration does not only take place from the poorer to richer countries, although that is an important pathway. Also within a country, there is a tendency that doctors move to urban areas from the periphery, to the private from the public sector, and to secondary from primary care.

## Why Do Physicians Migrate?

There are many factors that cause people to migrate. Among the push factors—stimulating to leave are low wages, poor job satisfaction and motivation, persistent shortage of basic supplies, dangerous working conditions, outdated equipment, lack of supervision and postgraduate training, limited career opportunities, and lack of employment opportunities. The pull factors are more or less the same with the opposite sign. Both push and pull factors are important for migrating health workers.

Mathauer and Imhoff interviewed health workers in Benin and Kenya, El Salvador and Nicaragua found that remuneration was not the main motivating factor for health workers (10). On the contrary, professional commitment/satisfaction and healing patients scored higher than remuneration in all these countries.

There is also a long process from thinking of leaving and to actually leave. Eke and coworkers surveyed medical students. In Hungary, 60–70% of all wanted to work abroad, but only 10–0% took active steps (11). Even fewer (2–3%) applied for recognition of diplomas abroad. In a similar study in Romania, the corresponding number was 10.2% of medical doctors who took such firm steps, and 3% actually moved.

Migrant health professionals are not a homogeneous group. The usual image is the “livelihood migrant”—the person who moves to earn a better life, and the “career oriented,” who moves to develop his or her career. But another group consists of “backpackers,” who works to travel, the “commuter,” who commutes across borders to work, the “undocumented,” who migrates for work, but unofficially and finally, the “returner,” who migrates in reverse (12).

People, including physicians, are affected by “the culturally constructed border.” They tend to seek to societies which resemble their own—defined by language, traditions, religion, feeling of belonging, cultural values, and so on. Examples are Moldovans moving to Romania, Belgians to the Netherlands and mobility within Scandinavia.

In Europe, during the last years, it has been very obvious that economy variations influence workforce. After the financial crisis in Greece, for instance, there has been an “exodus” of doctors. From 2010 to 2013, more than 4,000 doctors left Greece, more than 60% of whom were specialists who were “desperately looking for employment” (13).

Available data on the health workforce are fragmented. It can be difficult to separate out the effects of the crisis on the health workforce, and therefore to attribute causality to the impact of any specific economic and labor market changes on health workforce mobility.

Migration is complicated, and there are no simple solutions. It is a human right to move, and solutions must be found on a society and country level. Policy makers should concentrate on improving the pull factors and focus first on rural areas. All workforce planning has a lagged response, and we need to think out of the box to improve the situation.

The OECD has done some research on the drivers for mobility in Europe, and how it can be managed (14). They conclude that “there is a lack of systematic evidence about the relative effectiveness and cost-effectiveness of using the different instruments, and that further work is needed to understand what combination of policies that is best suited to motivating doctors.” They also found that it takes time to collect and analyze national and regional data, which often also is incomplete—some effects are lagged. It is difficult to generalize, as the workforce crisis affects countries in different ways, and there are important variations in how they have responded to the crisis.

## Code of Practice on International Recruitment

It is a well-known problem that rich countries drain poorer countries for their best brains, partly with the help of international recruitment agencies. Hence, the World Health Assembly adopted a “WHO Code of Practice on the international recruitment of health personnel” (15) in 2010. The goal was to “establish and promote voluntary principles and practices for the ethical international recruitment of health personnel and to facilitate the strengthening of health systems.” It stated that “Member States should discourage active recruitment of health personnel from developing countries facing critical shortages of health workers.”

In 2014, in view of the global economic crisis, WHO published a report on how the WHO code of Practice was implemented (16). Only 56; one-fourth of the Member States completed and returned the questionnaire, most of them from the European Section. At that time, 37/56 of them had made efforts to implement this code. Fifteen of the 56 were considering measures to change policies or laws on international recruitment of health personnel and only 9/56 encouraged and promoted laws or good practices among recruitment agencies. There were major variations in how migrants and domestic health personnel enjoyed legal rights and responsibilities, remuneration, training opportunities, and being allowed to make timely and informed decisions on their employment positions. In addition, there were major flaws in how countries kept statistical records on the number and qualifications of migrant health personnel.

This exercise was repeated in 2016, and now, the findings were less depressive (17). This time, 74 countries replied, and 70% of Member States stated that domestically trained health personnel and migrant health personnel have the same legal rights and responsibilities. Two-thirds of countries have undertaken measures to retain, sustain, and educate domestic health workforce, 58% of the countries have adopted measures to minimize geographical maldistribution and also to improve retention in areas that are underserved. Twenty-four percent of Member States that responded consider changing laws/policies into a form compatible with the Code recommendations. Yet, there is a long way to go.

## Retaining Physicians

We have seen that migration is a complex interaction of market forces, the workplace, and the individual worker. It is a human right to move, and maintaining and developing the workforce locally or within a country cannot be an individual responsibility of health personnel. WHO has recommended action at three levels (17):

1. Source countries must strengthen health workforce retention. There seems to be a link between internal and international migration. Therefore, they should concentrate on rural areas. In those areas, local workers should be trained in the local language and the content should be relevant. Even if there is continuing migration, they should expand training. Employment opportunities for graduates must also grow. The success depends on involvement and support as well as on-the-job-incentives of key institutions (hospitals and universities). It must be easy to return home after working abroad or in central locations. Local conditions must be improved—financially and good management, safety and career development must be provided. Living conditions should be improved, including transport, housing, and education of family members. Bonding central–rural institutions is also a possibility.
2. Receiving countries must adhere to ethical recruitment standards. They should provide support to human resources in source countries, directly targeted to expand workforce, medical brigades of direct twinning of health institutions. They should ensure fair treatment of migrants and give them terms and conditions equal to local staff, teach them in their rights, and have policies concerning racism. One should also keep in mind that receiving countries are often source countries, as well.
3. International instruments are not legally binding but set important norms for behavior. Bilateral agreements are complicated and have shown limited impact, according to the WHO.

## Planning for the Future

Workforce planning is a complicated exercise. Several factors must be taken into account, and migration is just one of them (14). The patient population—the number, sickness panorama, and patients’ expectations, is one factor. The society has a certain level or aspiration of the quality and the quantity of health care. The number and productivity of available health workers matters, as does the case health-care resources. The inflow and outflow of workers will vary, as will policies in education, remuneration, migration, and retirement. The age distribution of the workers affects planning. In the EU, the implementation of the European Working Time Directive regulates the number of hours a person is allowed to work (18). In addition, the younger generation is more inclined to deciding to spend more time with their families. Changing gender distribution in medicine might also play a role.

It becomes even more complicated when we take into account that all work force planning has a lagged response. It must relate to the effect of future technology/demand, the rate of growth of health expenditure, and unpredictable changes in migration flows, career change, and retirement. Often, there is no consensus on optimal organization model that will provide the most cost-effective use of a range of providers and their skills.

There might be a lack of cooperation on national and regional level. Many changes require more time than a short-lived political scene allows. Furthermore, carious stakeholders might undermine decisions. Nevertheless, planners must plan for the future based on the best available evidence.

## Predicting the Future in Anesthesiology

The distribution of the global workforce is maldistributed. Holmer and coworkers estimated the global surgical and anesthesia workforce (19). Forty-eight percent of the global population live in low and lower middle-income countries, but only 15% of anesthesiologists work there. The worst regions are Africa and South-East Asia. The World Federation of Anaesthesiologists represents 150 countries. Its mission is to improve patient care and access to safe anesthesia worldwide. The WFSA has recently undertaken a global workforce study and estimates that to fill the anesthesia workforce gap and concludes that more than 136,000 additional physician anesthesia providers are needed immediately to achieve a minimum density of 5 per 100,000 population in all countries (20). To achieve this standard, action is needed on country level. One example of a college of anesthesiology that has taken this seriously is Ireland. In 2014, the College of Anesthetists of Ireland, including the Pain Faculty, the national Clinical Program for Anesthesia, and the Joint Faculty of Intensive Care Medicine of Ireland, published a review of the manpower challenges in the country (21). This forecast, made by professionals, will help the Irish health authorities plan the future. Similar exercises should be performed in all countries. In the UK, the authorities used another approach when the Centre for Workforce Intelligence published their discussion documents for leaders in 2012 (22). They concluded that there would soon be too many anesthesiologists in the UK, something which was immediately countered by the Association of Anaesthetists of Great Britain and Ireland (23).

## Conclusion

Describing nationality and migration state is not as easy as one would think at first glance. Several factors are important when an individual decides to migrate, and remuneration is just one of them. Nations are not good at adhering to the agreed WHO Code of Conduct. Planning for the future is a complicated exercise, but all the unknown factors should not prevent professionals to take the lead. There is a huge demand for anesthesiologists in the world.

## Author Contributions

The author confirms being the sole contributor of this work and approved it for publication.

## Conflict of Interest Statement

The author declares that the research was conducted in the absence of any commercial or financial relationships that could be construed as a potential conflict of interest.

## References

[1] Egger Halbeis CB Cvachovec K Scherpereel P Mellin-Olsen J Drobnik L Sondore A Anaesthesia workforce in Europe. Eur J Anaesthesiol (2007) 12:991–1007. 10.1017/S0265021507000762 17608964

[2] OECD. International Migration Outlook 2015. Paris: OECD Publishing (2015).

[3] The European Observatory on Health Systems and Policies. In: Buchan J Wismar M Glinos IA Bremner J, editors. Health Professional Mobility in a Changing Europe. (2017). Available from: http://www.euro.who.int/__data/assets/pdf_file/0006/248343/Health-Professional-Mobility-in-a-Changing-Europe.pdf 29144691

[4] OECD. International Migration of Health Workers. (2010). Available from: http://www.who.int/hrh/resources/oecd-who_policy_brief_en.pdf

[5] International Bank for Reconstruction and Development/The World Bank. Migration and Remittances Factbook 2016, 3rd ed Washington, DC: International Bank for Reconstruction and Development/The World Bank (2016). Available from: https://openknowledge.worldbank.org/bitstream/handle/10986/23743/9781464803192.pdf

[6] WHO. The World Health Report 2006. (2006). Available from: http://www.who.int/whr/2006/06_chap1_en.pdf

[7] Chen L Boufford JI Fatal flows – doctors on the move. N Engl J Med (2005) 353:1850–2. 10.1056/NEJMe058188 16251543

[8] Global Knowledge Partnership on Migration and Development/The World Bank. Advance Edition, Migration and Remittances Factbook 2016 Third Edition. (2016). Available from: https://siteresources.worldbank.org/INTPROSPECTS/Resources/334934-1199807908806/4549025-1450455807487/Factbookpart1.pdf

[9] Masselink LE Government officials’ representation of nurses and migration in the Philippines. Health Policy Plan (2013) 1:90–9. 10.1093/heapol/czs028 22437505

[10] Mathauer I Imhoff I. Health worker motivation in Africa: the role of non-financial incentives and human resource management tools. Hum Resour Health (2006) 4:24. 10.1186/1478-4491-4-24 16939644 PMC1592506

[11] Eke E Girasek E Szócska M From melting pot to laboratory of change in central Europe. Hungary and health workforce migration. In: Wismar M Maier CB Glinos IA Dussault GJ, editors. Health Professional Mobility and Health Systems. Evidence from 17 European Countries. Copenhagen: World Health Organisation – European Observatory on Health Systems and Policies (2011). p. 365–94.

[12] Humphries N McAleese S Tyrrell E Thomas S Normand C Brugha R. Applying a typology of health worker migration to non-EU migrant doctors in Ireland. Hum Resour Health (2015) 13(52):1–12. 10.1186/s12960-015-0042-2 26111814 PMC4488134

[13] Tsolakidou S Greek Doctor Exodus, 4,000 Flee Country. GreekReporter.com (2013). Available from: http://greece.greekreporter.com/2013/01/31/greek-doctor-exodus-4000-flee-country/

[14] Simoens S Hurst J OECD Health Working Papers No. 21 The Supply of Physician Services in OECD Countries. Paris: OECD (2006). 11 p.

[15] WHO. WHO Global Code of Practice on the International Recruitment of Health Personnel. (2010). Available from: http://www.who.int/hrh/migration/code/code_en.pdf?ua=1

[16] Siyam A Roberto Dal Poz M Migration of Health Workers. Geneva: WHO (2017). Available from: http://www.who.int/hrh/migration/14075_Migration of Health_Workers.pdf

[17] WHO. Who global Code of Practice on the International Recruitment of Health Personnel. (2016). Available from: http://www.who.int/hrh/migration/infographic_EB2016_updt9may.pdf?ua=1

[18] European Union Law. Organisation of Working Time. (2016). Available from: http://eur-lex.europa.eu/legal-content/EN/TXT/?uri=LEGISSUM:c10418

[19] Holmer H Lantz A Kunjumen T Finlayson S Hoyler M Siyam A Global distribution of surgeons, anaesthesiologists, and obstetricians. Lancet Glob Health (2015) 3(Suppl 2):S9–11. 10.1016/S2214-109X(14)70349-3 25926323

[20] Kempthorne P Morriss W Mellin-Olsen J Gore-Booth J The WFSA Global Anesthesia Workforce Survey. Anesthesia & Analgesia (In Press). 10.1213/ANE.0000000000002258 28753173

[21] Centre for Workforce Intelligence. Providing Quality, Safe and Comprehensive Anaesthesia Services in Ireland – a review of Manpower Challenges. (2012). Available from: http://www.hse.ie/eng/about/Who/clinical/natclinprog/anaesthesia/Providing_Quality_Safe_and_Comprehensive_Anaesthesia_Services_in_Ireland.pdf

[22] Mannion D O’Sullivan E Foy F Golden M Golden B Smith J Shape of the Medical Workforce, Starting the Debate on the Future Consultant Workforce. College of Anaesthetists of Ireland (2014). Available from: https://www.gov.uk/government/uploads/system/uploads/attachment_data/file/507651/CfWI_future_consultant_workforce.pdf

[23] Redfern N Harrop-Griffiths W Who knows how many anaesthetists we need? Anaesthesia (2013) 3:227–31. 10.1111/anae.12171 23384255

